# An Investigation of Airborne Bioaerosols and Endotoxins Present in Indoor Traditional Wet Markets before and after Operation in Taiwan: A Case Study

**DOI:** 10.3390/ijerph18062945

**Published:** 2021-03-13

**Authors:** Da-Jiun Wei, Wen-Te Liu, Huin-Tsung Chin, Ching-Hsing Lin, I-Chun Chen, Yi-Tang Chang

**Affiliations:** 1 Department of Microbiology, Soochow University, Taipei 11102, Taiwan; g11111155@gmail.com; 2 Department of Tourism, Tungnan University, New Taipei City 22202, Taiwan; wdliu@mail.tnu.edu.tw; 3 The Graduate School of Technology for Hazards Mitigation, Tungnan University, New Taipei City 22202, Taiwan; fire70214@gmail.com; 4 Center of General Education, National Taitung College, Taitung 95045, Taiwan; chlin@ntc.edu.tw; 5 Department of Land Resources, Chinese Culture University, Taipei 11114, Taiwan

**Keywords:** indoor traditional wet market, bacterial bioaerosols, fungal bioaerosols, chemical sanitization, endotoxin

## Abstract

Customers in Taiwan prefer to purchase fresh foods and household supplies at indoor traditional wet markets (TWMs). The health risk to indoor TWM staff exposed to bioaerosols needs to be evaluated, since these workers spend long periods of time in the market for stall preparation, selling, and stall cleaning. This study investigated the bioaerosols present in two indoor TWMs. The results showed that the cleaning process at Market A after operations, involving the use of an agitated waterspout, was able to decrease the concentration of bacterial bioaerosols (BBs) by an average of 64%, while at the same time increasing the concentration of fungal bioaerosols (FBs) by about 2.4 fold. The chemical sanitization process at Market B after operations was able to bring about average decreases of 30.8% in BBs and 19.2% in FBs, but the endotoxin concentration increased. Hotspots were found to be associated with vendors of fresh, live poultry and fresh, raw meat/seafood. *Pseudomonas* spp. and *Clostridium* *perfringens*, both of which can be pathogenic, were found to be the dominant species present in these markets, making up 35.18% to 48.74% and 9.64% to 11.72% of the bacteria present, respectively. Our results provide fundamental information on the distributions of bioaerosols and endotoxins within indoor TWMs both before and after operation.

## 1. Introduction

Bioaerosols, defined as airborne particles from biological sources, include pollen, bacteria, fungal spores, and viruses [1]. Airborne bioaerosols are easily transported through the air and are able to propagate rapidly under conditions of high relative humidity (70% to 90%) found in areas with a subtropical monsoon climate, such as in Taiwan [2,3]. The impacts of environmental factors, (such as temperature, relative humidity, land use, human behavior, and particle size) on bioaerosols formation are complex [3,4,5]. High concentrations of bioaerosol are common around biological sources such as animal farms and composting sites [6,7,8]. It has been reported that points of interest of bioaerosols include farms, pastures, gardens, garbage dumps, schools, and fuel stations [8,9]. Furthermore, bioaerosols pose a threat to human health and can result in various infectious diseases, toxicity, and hypersensitivity [10]. Bacterial bioaerosols (BBs) and fungal bioaerosols (FBs), which range in size from 0.1 to 10 μm (aerodynamic diameter), can be inhaled directly into the respiratory system [11]. Studies have shown that when the human body is exposed to a high concentration of bioaerosols for a long period of time, lung dysfunction and severe systemic inflammatory responses occur, such as pneumonia, asthma, rhinitis, and various respiratory infections [6,12,13,14]. A number of vulnerable groups, such as children, young adults, the elderly, those suffering from chronic respiratory diseases, and those suffering from cardiovascular diseases, may be harmed if exposed to high concentrations of bioaerosols [6,15]. Endotoxins are typically released after cell lysis or during active Gram-negative bacteria cell growth and they form the primary non-culturable component of airborne bioaerosols [16,17,18,19]. People are usually exposed to airborne endotoxins because these molecules have become attached to the surfaces of larger particles, either PM_2.5_ or PM_10_ particles, leading to immune functioning disorders as well as acute/chronic inflammation of the lungs [17]. The incidence of individuals with such diseases increases significantly with an increase in exposure time due to spore endotoxin production during bacterial growth in a variety of different environments [13,14,17,20]. The exposure of high-risk groups to a high concentration of FB mycotoxins can increase the symptoms associated with allergies and asthma. Specific respiratory tract-related diseases, such as allergic bronchopulmonary mycoses, allergic fungal sinusitis, and hypersensitivity pneumonitis, are often observed among workers [21,22]. Furthermore, synergistic interactions between biological bioaerosols and chemical air pollutants also may increase the risk of respiratory disease [23]. Bacterial bioaerosols have been associated with PM_2.5_ and are monitored using a range of particles with diameters from 0.5 to 5 μm; particles in this range can be either BBs or FBs. Such BB/FB PM_2.5_ particles are considered a major cause of various allergies and respiratory diseases [24].

People spend the majority (nearly 90%) of their time indoors due to modern lifestyles and social patterns. Indoor air quality has become an important environmental issue because the concentration of air pollutants in an indoor environment is usually higher than that found in an outdoor environment. Indoor BBs/FBs of various types usually exist in indoor high occupancy areas and can also be transported as bioaerosols from the outdoors [16,25]. Variations in indoor airborne bioaerosols are often influenced by different seasons. The indoor vs. outdoor ratio of BBs in high-density population areas, such as schools and kindergartens, is over 1 [26,27]. Long-term exposure to indoor areas that have inadequate air exchange and poor air quality may result in sick building syndrome, allergic reactions, respiratory tract infections, and lung cancer [16,26]. A previous study indicated that, when there is a significant infectious disease risk present, sick building syndrome is frequently observed when airborne BB counts reach a concentration of 10^2^–10^3^ CFU m^–3^ [26]. Studies have shown that fungal growth has a strong, positive relationship with increases in relative humidity and temperature. The Taiwan EPA stated that the concentrations of formaldehyde, total volatile organic compounds, PM_10_, PM_2.5_, O_3_, bacteria, and fungi in a given indoor space should be below the those defined in the Taiwan Indoor Air Quality Standard (IAQS). Based on the Taiwan IAQS requirement, the users (or the owner) of an indoor environment are requested to develop a detailed plan for regular monitoring of IAQS air pollutants, and they also need to develop an effective cleaning process in order to maintain good air quality. This includes the control of BBs and FBs in relevant indoor spaces. In other eastern Asian countries with the same living habits, such as Hong Kong and Singapore, the guideline (or standard) for BB is <500 CFU m^−3^, which is much lower than that stated in the Taiwan IAQS (BB < 1500 CFU m^−3^). This is likely to expose individuals at indoor markets to bioaerosol levels that put them at risk of infection, allergy, and other consequences.

People in Taiwan prefer to purchase their daily supplies at traditional wet markets (TWMs) because they are able to bargain with vendors about price. Vendors sell a wide range of different types of goods based on their customers’ needs in a particular neighborhood; these include fresh raw meat, fresh uncooked seafood, fruit, vegetables, various cooked foods, general merchandise, cloth/material, and a range of other dry goods. They usually open early and close at about noon every day. Historically TWMs are found adjacent to residential areas and cover several streets; they are located in the semi-open overhangs of storefronts along the pavement, and this often results in poor sanitation and the production of bioaerosols that contain both bacteria and fungi; these are able to interact with vendors and customers both indoors and outdoors [20]. The BBs/FBs that form the indoor microbial community are present at different levels depending the human activities taking place [28]. Thus, the distribution of BBs and FBs within different traditional markets varies on a case-by-case basis and is dependent on their locations within the market and the goods being sold. For example, members of γ-Proteobacteria, including *Acinetobacter calcoaceticus*, *Pseudomonas luteola*, *Pseudomonas mendocina*, *Pseudomonas multophila*, *Serratia plymuthica*, *Escherichia coli*, *Yersinia intermedia,* and *Providencia* spp., have been found to be present in markets selling vegetables in India [13]. Poultry manure is known to be an important source of bioaerosols in wet markets; these bioaerosols can often include pathogenic bacteria and organisms that encode antibiotic resistance genes. Thus, they play an important route in the transmission of microbial contamination [29]. In terms of specific bacteria present in traditional markets, Gram-positive *Streptococcus* spp. have been identified as associated with pork meat sold by retailers [30,31,32]. Furthermore, at a traditional Hong Kong market, it was found that the concentrations of bacteria and PM_10_ in the poultry district were twice as high as those in the livestock meat district and the seafood district [20]. 

Recently, more indoor public TWMs have been set up by city governments in commercial buildings with the aim of providing better control of air quality during their operation. Market staff are usually required to set up an autonomous management committee at a public TWN, but many of these markets have not established complete management mechanisms for the control of ventilation, daily cleaning, disinfection, etc. Indoor public TWMs have been specifically targeted in terms of their workplace biological hazards due to the presence of densely crowded footpaths and the fact that there is close contact between staff/customers. Market staff are a major concern in these enclosed spaces, since they are present in these TWMs for a much longer time than customers are. They are present from when they begin to prepare their stall before opening for business until they clean up their stall after closure. Taking all of the above into account, it is necessary to improve the understanding of the distribution of airborne bioaerosols in these markets in order to explore the occupational safety and health needs of the staff who work in these indoor public TWMs in Taiwan. In the present preliminary study, for the first time, the culturable organism counts of BBs and FBs of different sizes in Taiwan are assessed before operations have begun at TWMs and after operations have finished at TWMs. The bacterial community structure and the presence of airborne endotoxins released from Gram negative bacteria are also analyzed at hotspots within the two markets studied after operations are over. However, this study has some limitations. In this study, we only focus on two TWMs and only compare the bioaerosol concentration before and after the operation of these markets. The current results thus lack time-series data for these two markets, and only a limited number of study locations are described. Moreover, this study ignores the fact that indoor FB species may not always reflect the species composition outdoors because of air dilution due to the large air spaces in such buildings. More experiments, such as measurement of dominant FB species and their mycotoxins, should be conducted. The indoor vs. outdoor ratio of FBs needs to be measured. Due to a lack of information on the complex distribution of pipes and outlets on the ceiling to supply fresh air, we evaluate how the overall performance of different ventilation systems affects the distribution of BBs and FBs in indoor TWMs.

## 2. Materials and Methods 

### 2.1. Indoor Traditional Wet Markets

In order to avoid possible effects due to the different attributes of the buildings, studied such as the age of the building, building materials used, the variation in indoor vs. outdoor environmental conditions (temperature, humidity, and wind speed), the size of each TWM, all of which will influence the indoor BBs and FBs and the microbiome present in a building, two indoor public TWMs: Market A and Market B, both of which are located in Taipei City, were selected for this study. Table 1 lists the diverse products sold in the ten districts and the basic information about each market. These products included fresh poultry raw meat, fresh livestock raw meat, fresh raw seafood including fish, vegetables, fruit, and grains, together with chicken eggs and various other goods. 

### 2.2. Analysis of Airborne BBs and FBs

**Figure 1:**
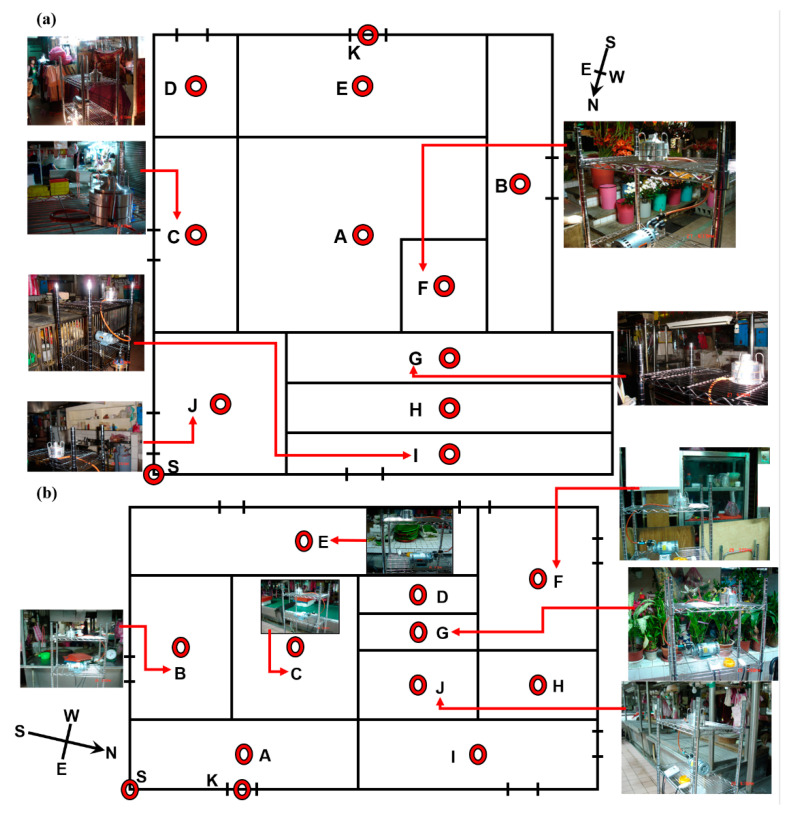
Bioaerosols present at the sampling locations (red circular dots) collected on the footpaths of the ten different districts; (**a**) Market A and (**b**) Market B.

The aerobic plate counts of airborne BBs and FBs present in the indoor TWMs were analyzed using the culture-based methods of Taiwan’s EPA, namely the “Detection method of bacterial concentration in the air (NIEA E301.10C)” and the “Detection method of fungal concentration in the air (NIEA E401.15C)”, respectively. The sampling locations on the major footpaths of various districts are shown in Figure 1 and Table 1, and these are classified according to the goods being sold. Entrances to the indoor TWMs were selected as the outdoor sample areas (control). A two-stage viable Andersen Cascade Impactor (ACI) was used to collect airborne bioaerosols for one hour at each sampling location before the start of market operation (time: 03:00 to 04:00) and after the market had closed (time: 13:00 to 14:00) (Table 2). The operation is defined as the market is opening that allows customers to purchase products on stalls. Each stage in the ACI consisted of uniformly distributed precision drilled holes; these were 1.18 mm in diameter for the first stage and 0.25 mm in diameter for the second stage and were used to collect two different sizes of particles: (1) ≧8.0 μm bioaerosols (defined as large particle bioaerosols, LPBs); (2) 1.0–8.0 µm (defined as small particle bioaerosols, SPBs), respectively. The ACI was placed at a height of 150 cm above the floor surface at the sampling locations in each of the districts being tested; this is where the human breathing zone is situated. The flowrate of the ACI during the collection of the bioaerosol samples was set at 28.3 L min^−1^ by adjusting the rate using dry flow calibration equipment. The agar media used were soybean casein digest agar for the growth of bacteria and malt extract agar for the growth of fungi. The bioaerosol sampling time was determined using precise timers designed for our bioaerosol collecting equipment (see Figure 1), and the equipment was set up so that 30–300 CFU m^−3^ colonies were collected on a standard 9-cm Petri dish. The ACI was completely sterilized using 70% ethanol for each sampler before each sampling period; this was to avoid the air inside the ACI forming a major part of the collected sample. Sampling was carried out in triplicate at each sampling location. Bacteria and fungi that had landed on the plates were incubated at 30 ± 1 °C for 48 ± 2 h, or 25 ± 1 °C for 5 ± 2 d, respectively. Table 2 shows the indoor vs. outdoor environmental parameters, including temperature, relative humidity, and wind speed, that were present during sampling at the indoor vs. outdoor TWMs. The contour plot of the bioaerosol concentration distributions within the TWMs was analyzed using contour lines with Surfer12 software.

### 2.3. Analysis of Bacterial Community Structure at Hotspots

A rapid and sensitive diagnostic tool, fluorescence in situ hybridization (FISH), was used to determine the bacterial community structure [33]. This analysis was carried out at hotspots within each TWM after closure, which is when they were found to have the highest concentrations of BBs. Table 3 details the 16S and 23S rRNA oligonucleotide probes used. These consisted of (1) a domain probe for bacteria; (2) three phyla probes for Gram-negative bacteria that targeted α-Proteobacteria, β-Proteobacteria, and γ-Proteobacteria; (3) two phyla probes for Gram-positive bacteria that targeted Actinobacteria and Firmicutes; (4) three species-specific probes targeting *Pseudomonas* spp., *Streptococcus* spp., and *Clostridium perfringens*; and (5) a negative control probe (NONEUB338) for detecting nonspecific hybridization. Details of the oligonucleotide probes are available at the probeBase website [34]. The probes were labeled with a CY3™ fluorescent tag at the 5′ end. The experimental FISH procedure began by adding the collected hotspot samples into 20 mL of sterile solution using a biosampler^®^ at a flowrate of 12.0 L min^−1^ for 30 min. The filtered samples then underwent cell membrane lysis. This was followed by fixing the rRNA onto gelatin-coated slides. After hybridization with the FISH probes, the remaining probes in the samples were removed by washing. The bacterial cells were then visualized by fluorescence microscopy.

The microscope was equipped with a digital camera and Image-Pro Plus software (Version 6.0). The total cell count was 500–1500 bacteria per sample. The microscopic analysis included manual counting of the cells from at least ten photographs of the duplicate samples to assess the quality control of the procedures. The average cell counts and their standard deviations were determined by counting bacterial numbers and their impurities. 

The microscope was equipped with a digital camera and Image-Pro Plus software (Version 6.0). The total cell count was 500–1500 bacteria per sample. The microscopic analysis included manual counting of the cells from at least ten photographs of the duplicate samples to assess the quality control of the procedures. The average cell counts and their standard deviations were determined by counting bacterial numbers and their impurities. 

### 2.4. Endotoxin Analysis

Endotoxin-lipopolysaccharide is a representative biomarker that can designate a characteristic group of constituents present in the outer membranes of Gram-negative bacteria. Endotoxin analysis at the various hotspots within the indoor TWMs after closure was measured using the Limulus amebocyte lysate (LAL) test, which is a rapid, sensitive, and highly standardized test [35]. During the LAL test, Gram-negative bacteria are detected via a series of reactions in which the endotoxin catalyzes the activation of coagulase. The concentration of endotoxin is determined by the initial rate of activation. Samples were initially collected for 2 h at a 2.0 L min^−1^ flowrate onto polycarbonate filter paper (0.4 µm pore size and 37 mm diameter) in a polystyrene cassette. Pyrogen-free water (LAL Reagent water) was then added to each sample using pyrogen-free tips on filter paper which had been placed in a pyrogen-free tube. Aqueous samples were then prepared by vortexing, which was followed by ultrasonic extraction for 1 h. The LAL test reagent is formulated using a synthetic substrate and produces a chromophore when cleaved by the endotoxin-activated enzyme. The LAL test uses chromogenic endotoxin testing reagents with a specific buffer and is read at 405 nm using a microplate reader after incubation at 37 °C for 23 min. The calibration standards for the concentration of endotoxin gave a good linear equation with *r*^2^ = 0.966 (data not shown), and the control standard endotoxin used was a purified extract of *E. coli* O113: H10, 3.13 EU ng^-1^ and covered the range 0.005–1.28 EU mL^−1^.

## 3. Results

### 3.1. Concentration of Bioaerosols in Market A

**Figure 2:**
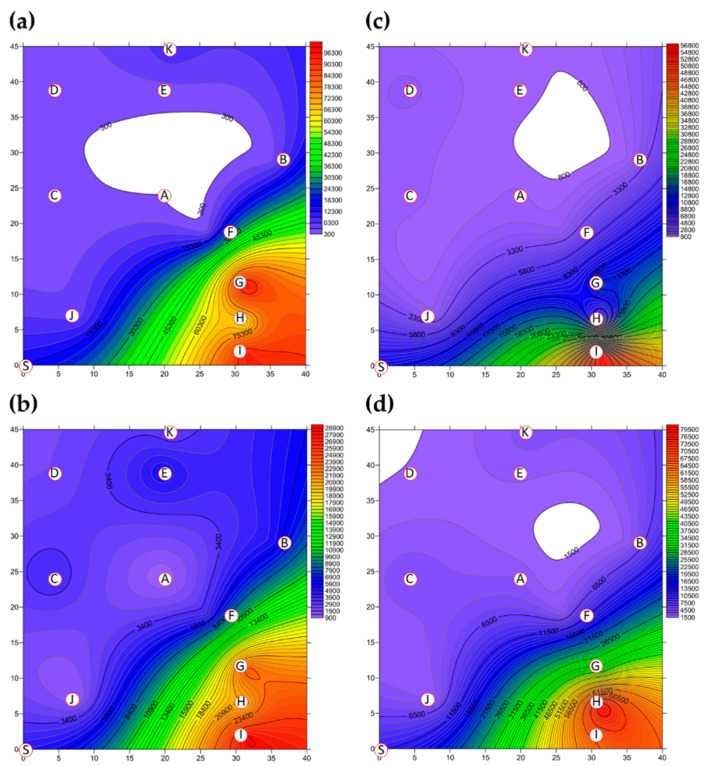
The total bacterial bioaerosol (BB) concentration (CFU m^−3^) present in Market A (**a**) before operations commenced and (**b**) present after operations had finished; the total fungal bioaerosol (FB) concentration (CFU m^−3^) in Market A (**c**) before operations commenced and (**d**) present after operations had finished. Red circular dots with English titles represent the sampling locations. Bioaerosol levels in the areas marked in white were below the cutoff values.

**Figure 3:**
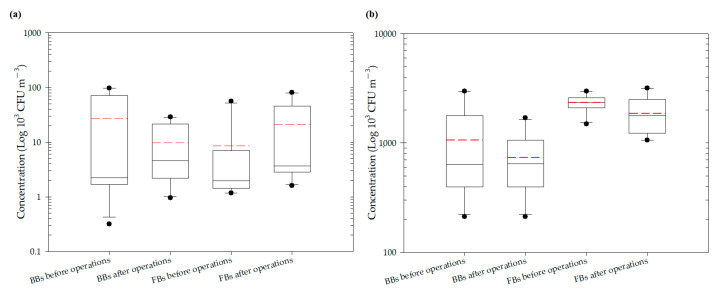
Average total bioaerosol concentrations in the two indoor TWMs: (**a**) Market A; (**b**) Market B.

A two-dimensional representation of the BBs present in Market A is shown in Figure 2. The total concentration of BBs present in Market A before operations commenced is shown in Figure 2a. Hotspots, together with the percentage of total BBs, consisted of the livestock fresh raw meat district (District A-G, 36.08%), the live poultry and fresh raw meat district (District A-I, 34.95%) and the fresh raw seafood district (District A-H, 23.34%), as shown in Figure S1. The concentrations of BBs in eight of the districts were above the values outlined in the Taiwan IAQS (<1.5 × 10^3^ CFU m^−3^), while the fruit and vegetable district (District A-A) and the grain district (District A-D) were below the given values. The average concentration of BBs for the ten districts before the start of market operations was 2.71 × 10^4^ CFU m^−3^, as shown in Table 3 and Table S2. The average concentration of BBs in the three hotspots was 8.53 × 10^4^ CFU m^−3^, which made up 94.37% of the total BBs. The total BB concentrations present after operations had finished are shown in Figure 2b. The highest concentrations and percentages of total BBs consisted were found in the live poultry and fresh raw meat districts (Districts A-H): 2.86 × 10^4^ CFU m^−3^ and 21.46%. The average concentration across all districts after closure decreased to 9.68 ×10^3^ CFU m^−3^, as shown in Figure 3 and Table S2, which is 64% of that present in the market before operation. The average concentration of the three hotspots was higher, 2.44 × 10^4^ CFU m^−3^, after operations had finished; this represented 75.81% of all BBs and a reduction of 29% compared to the level before the start of market operations.

The two-dimensional distribution of FBs in Market A is shown in Figure 2. The total FB concentration present in Market A before operations commenced is shown in Figure 2c. The average concentration of FBs across all ten districts before market operations started was 8.63 × 10^3^ CFU m^-3^, as shown in Figure 3 and Table S2. The high-concentration hotspots in terms of the percentages of all FBs were the live poultry and fresh raw meat district (District A-I, 64.99%), the livestock fresh raw meat district (District A-G, 16.46%), and the fresh seafood district (District A-H, 5.28%), which are the same hotspots as for BBs. The average concentration in these hotspots was 2.49 × 10^4^ CFU m^−3^. The concentrations in hotspot district A-I and district A-G were higher than the Taiwan IAQS values (<1.0 × 10^3^ CFU m^−3^ and the ratio of indoor FBs/outdoor FBs <1.3). The total FB concentration present after operations had finished is shown in Figure 2d. However, the concentration of FBs after market operations had finished did not decrease, even after the purchasing activities of the customers had finished and the clean process has been executed by each vendor. In fact, the average concentration after market closure was found to have increased to 2.09 × 10^4^ CFU m^−3^ (see Figure 3 and Table S2), which is an increase of 2.4-fold compared to before market operations started. The concentrations at the hotspots (Districts A-G, A-H and A-I) were higher than the Taiwan IAQS (see Figure S2). The concentration at the fresh raw seafood hotspot (A-H district) after market operations had stopped increased by almost 17.5-fold compared to the concentration before the market started. The percentage of FBs also increased from 5.28% before operation started to 38.44% after market closure. Finally, the concentration of FBs in the poultry fresh raw meat hotspot (District A-I) also increased, but in this area, the percentage of FBs decreased from 64.99% before market operations began to 32.52% after market operations finished.

### 3.2. Concentrations of bioaerosols in Market B

**Figure 4:**
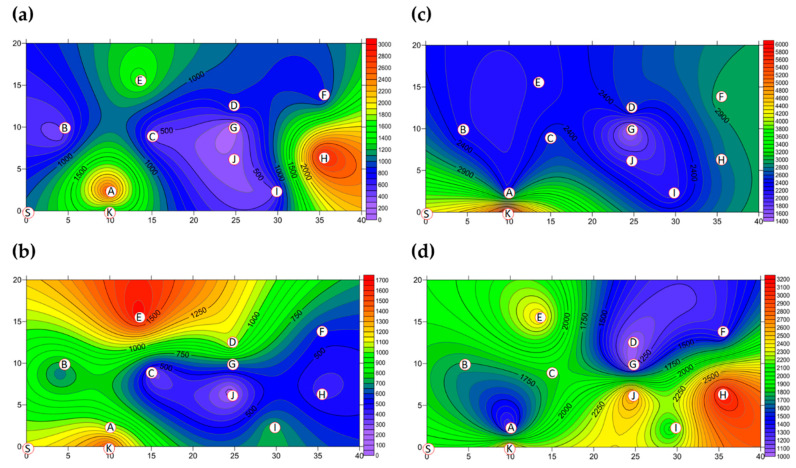
The concentration (CFU m^−3^) of total BBs present in Market B (**a**) before operations commenced and (**b**) after operations had finished; the total FB concentration in Market B (**c**) before operations commenced and (**d**) after operations had finished. Red circular dots with English titles show sampling locations.

The two-dimensional distribution of BBs in Market B is shown in Figure 4. The total BB concentration present in Market B before operations commenced is shown in Figure 4a and Figure S2. The hotspots with their percentages of total BBs were the grocery district (District B-H, 28.00%) and the grain district (District B-A, 25.00%), which were measured to be 2.65 × 10^3^ CFU m^−3^ and 2.96 × 10^3^ CFU m^−3^, respectively, while bioaerosols in the other eight districts were within the levels set by Taiwan’s IAQS. The average concentration across all districts of Market B was 1.06 × 10^3^ CFU m^−3^ before operating, as shown in Figure 3 and Table S2, which was 10 times less than that in Market A. Moreover, the total BB concentration present after operations had finished is shown in Figure 4b. The average concentration of BBs across all districts before market operations began was found to be similar to the concentration aftermarket closure, as shown in Figure 3 and Table S2. The average concentration for the hotspots after operations had been completed was lower at 7.34 × 10^2^ CFU m^−3^, a reduction of 31% compared to that before market operations began. There were lower levels of BBs in the hotspots after market operation had finished, such as the concentrations in the five districts B-B, B-D, B-E, B-G, and B-I, which were slightly higher when compared to the concentrations before market operations began (see Figure S2). For example, in the livestock fresh raw meat district (District B-D), the bioaerosol level of 8.48×10^2^ CFU m^−3^ before market operations commenced and this increased to 1.06 ×10^3^ CFU m^−3^ after market operation had finished. Similarly, the poultry fresh raw meat district (District B-B) had a concentration of 4.24 × 10^2^ CFU m^−3^ before market operations began and 6.60 × 10^2^ CFU m^−3^ after market operations had finished.

The two-dimensional distribution of FBs in Market B is shown in Figure 4. The total FB concentration present in Market B before operations commenced is shown in Figure 4c and Figure S2. The average concentration of FBs was found to be 2.33 × 10^3^ CFU m^−3^ across all ten districts before market operations commenced, as shown in Figure 3 and Table S2, which is lower than the control value (6.04 × 10^3^ CFU m^−3^) at the entrance to the market (District B-K). Although the concentrations of FBs in all districts were higher than 1 × 10^3^ CFU m^−3^, they complied with the Taiwan IAQS (https://law.moj.gov.tw/Eng/index.aspx; accessed on 23 November 2011.) because the indoor/outdoor ratio was less than 1.3. The reason for the unusual FB indoor/outdoor ratio obtained during this study, which was usually low, is that Taiwan is a country with a high relative humidity and warm temperatures for most of the year; these factors result in rapid fungal propagation and large-scale spore germination [36,37]. High-concentration hotspots with the percentage of all FBs were in the following order (See Figure S2): 2.96 × 10^3^ CFU m^−3^ for the cooked food district (District B-F, 12.73%); >2.75 × 10^3^ CFU m^−3^ for the grocery district (District B-H, 11.82%); and >2.54 × 10^3^ CFU m^−3^ for the seafood fresh raw meat district (District B-C, 10.91%) and the livestock fresh raw meat district (District B-D, 10.91%). The total FB concentrations present after operations had finished are shown in Figure 4d. The average concentration of FBs across the districts after operations had been completed decreased slightly to 1.86 × 10^3^ CFU m^−3^, as shown in Figure 3 and Table S2, which was 80% of the value present before market operations commenced. Concentrations of FBs at the three districts B-E, B-H, and B-J were slightly higher when compared with those before market operations began. The three hotspots had concentrations in the following order: 3.18 × 10^3^ CFU m^-3^ for the groceries district (District B-H, 17.09%); >2.65×10^3^ CFU m^−3^ for the livestock meat district (District B-J, 14.24%); and >2.44 × 10^3^ CFU m^−3^ for the vegetable and fruit district (District B-E, 13.10%) (Table S2 and Figure S2).

### 3.3. The Size Distribution of Bioaerosol Particles in TWMs 

**Figure 5:**
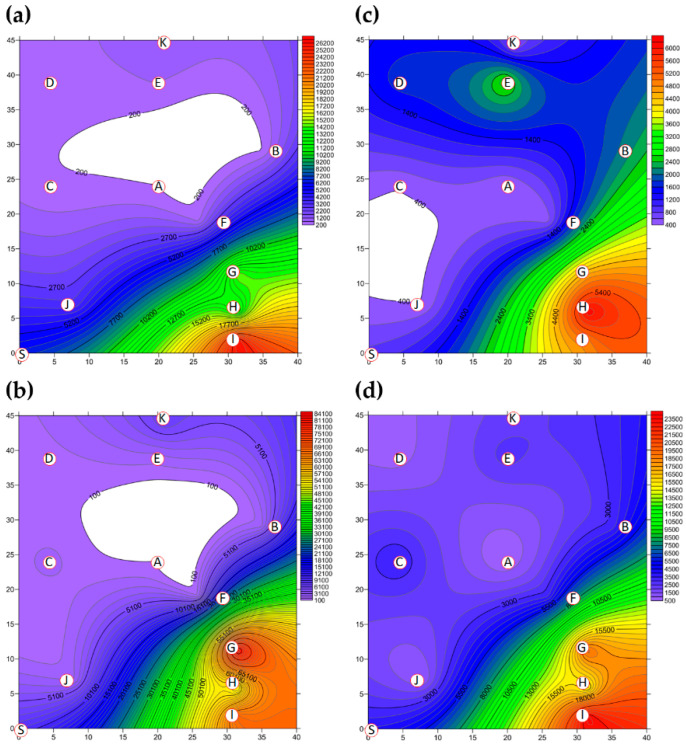
The bacterial bioaerosols (BBs) (CFU m^−3^) present as (**a**) LPBs and (**b**) SPBs in Market A before operations commenced and the concentrations of (**c**) LPBs and (**d**) SPBs present after operations had finished. Red circular dots with English titles show sampling locations. Bioaerosols in the white area had values below the scale.

**Figure 6:**
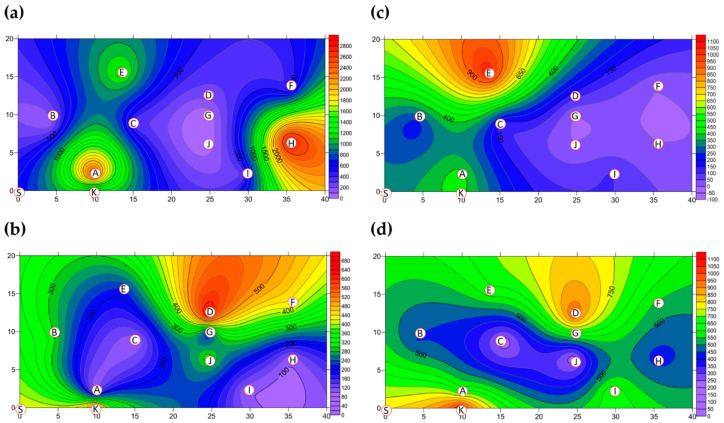
The BBs (CFU m^−3^) present as (**a**) LPBs and (**b**) SPBs in Market B before operations commenced and (**c**) LPBs and (**d**) SPBs present after operations had finished. Red circular dots with English titles show the sampling locations.

**Figure 7:**
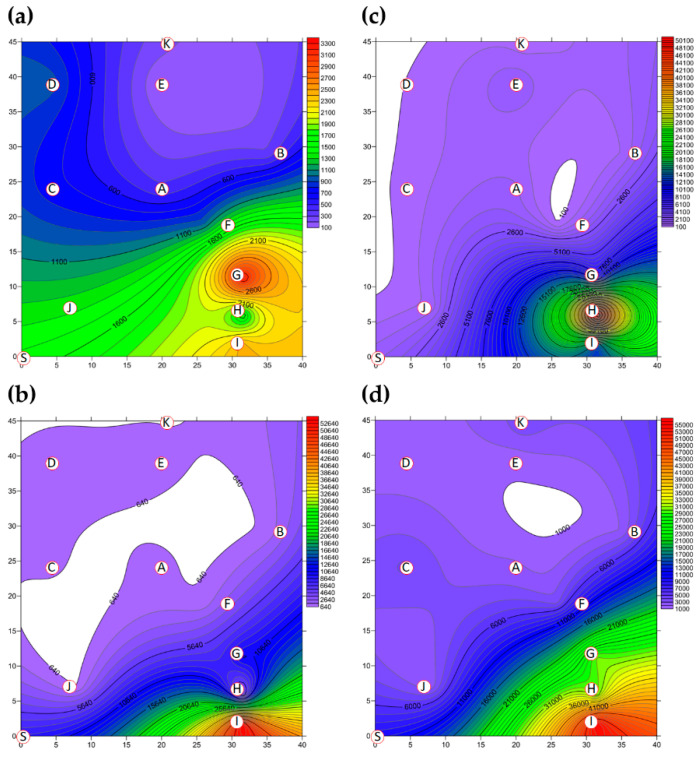
The FBs (CFU m^−3^) present as (**a**) LPBs and (**b**) SPBs in Market A before operations commenced and (**c**) LPBs and (**d**) SPBs present after operations had finished. Red circular dots with English titles represent the sampling locations. The bioaerosol levels in the areas marked in white are below the cutoff level.

**Figure 8:**
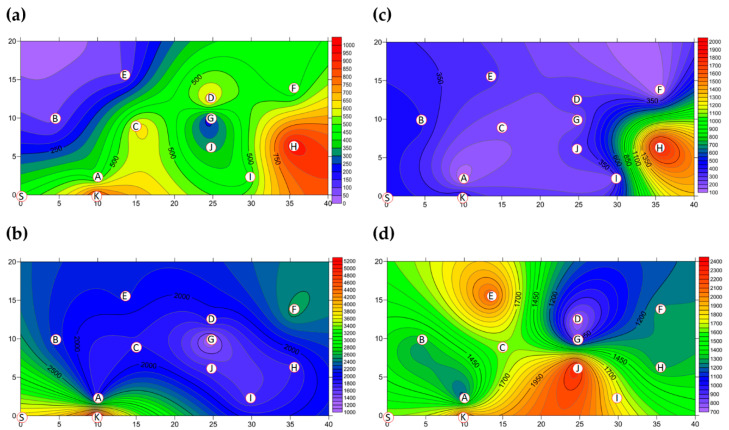
The FBs (CFU m^−3^) present as (**a**) LPBs and (**b**) SPBs present in Market B before operations commenced and (**c**) LPBs (**d**) SPBs present after operations had finished. Red circular dots with English titles represent the sampling locations.

The two-dimensional distributions of bacterial LPBs and SPBs in Market A before operations commenced and after operations had finished are shown in Figure 5, respectively. There was significant difference (*p* < 0.05) in LPB and SPB concentrations before and after the market operation (data not show). The size distributions of BBs in the two indoor TWMs before operations started and after operations finished are shown in Table 4, respectively. In Market A, the percentages of bacterial SPBs in districts were higher than the percentages of bacterial LPBs (except for District A-H). The percentages of bacterial LPB and SPB in Market A were measured at 22% and 78% before operation, respectively, and 25% and 75% of the concentrations after finishing, respectively. Most bacterial SBP in Market A before operations started (see Figure 5b) and after operations finished (see Figure 5d) were present in three hotspots (Districts A-G, A-H, and A-I), all of which had high bioaerosol levels. By way of contrast to the above findings, the two-dimensional distributions of bacterial LPBs and SPBs in Market B before operations commenced and after operations had finished are shown in Figure 6, respectively. Table 4 shows that the percentages of bacterial LPB and SPB present in Market B were significantly different before operations started and after operations had finished, respectively. The percentages of LPB before operations started were 76% and 24%, respectively. Figure 6a shows high levels of LPBs were found in three districts: 2.54 × 10^3^ CFU m^−3^ in the groceries district (District B-H), 2.54 × 10^3^ CFU m^−3^ in the grains district (District B-A), and 1.27 × 10^3^ CFU m^−3^ in the vegetable and fruit district (District B-E). After market operation, the percentages of bacterial LPB and SPB present in Market B changed obviously to 27% and 73%, respectively (Table 4). The bacterial SPB concentration increased from 2.54 × 10^3^ before market opening to 5.32 × 10^3^ after operations in Market B because some districts were still undergoing cleaning (e.g., District B-D, District B-E) or were open (e.g., District B-G, District B-I) without air conditions. Figure 6b shows there were no bacterial LPBs (0 CFU m^−3^ LBP) in four districts, namely the cooked food (District B-F), flowers (District B-G), groceries (District B-H), and livestock fresh raw meat (District B-J) districts. The highest concentration of bacterial SPBs was found to be 9.54 × 10^2^ CFU m^−3^ in the livestock fresh raw meat district (District B-D). The two-dimensional distributions of fungal LPBs and SPBs in Market A before operations commenced and after operations had finished are shown in Figure 7, respectively. Table 4 shows the average concentrations of fungal LPB and SPB that were generally present for both indoor TWMs. The average percentage of fungal SPBs in Market A was 86% before operating and 63% after finishing. The concentration of fungal SPB in all districts of Market A after operations had been completed (1.33 × 10^4^ CFU m^−3^) was higher compared with that before operations began (7.42 × 10^3^ CFU m^−3^). Furthermore, the two-dimensional distributions of fungal LPBs and SPBs in Market B before operations commenced and after operations had finished are shown in Figure 8, respectively. A similar assessment of fungal SPBs in Market B showed that the percentage was 82% before operations began and 78% after operations had been completed (Table 4). The level of fungal SBPs decreased slightly from 1.91 × 10^3^ CFU m^−3^ before operations began to 1.45 × 10^3^ CFU m^−3^ after operations finished. Nevertheless, the fungal SBP concentration increased to 2.32 × 10^3^ CFU m^−3^ in the livestock fresh raw meat district (District B-J) and to 1.08 × 10^3^ CFU m^−3^ in the general merchandise district (District B-I) (see Figure 8d), possibly due to there being more/excess water and/or excess organic material being present.

### 3.4. Bacterial Community Structure of Bioaerosols in Hotspots

Table 5 shows the bacterial community structures after operations had finished at the two TWMs for the two hotspots that had the highest levels of BBs: The A-G livestock fresh raw meat district and the B-H groceries district. The *Bacteria* domain made up similar percentages: 74.37 ± 4.90% in District A-G and 70.87 ± 5.10% in District B-H. Furthermore, the five phyla investigated in this study made up 96.66% of the organisms present in District A-G and 69.61% of the organisms in District B-H. β-Proteobacteria, Actinobacteria, and Firmicutes were dominant with a range of 53.93% to 69.98% for the two TWMs. A trend toward a higher abundance of Firmicutes (>59.2%) within the pig and poultry farm buildings was detected. The total percentages of the three Gram-negative α, β, and γ-Proteobacteria phyla were 61.43% for District A-G and 32.79% for District B-H. Three specific *Pseudomonas* spp. formed a high percentage of the bacteria present in the TWM hotspots after operation had finished, being dominant in District A-G (48.74%) and District B-H (35.18%). The level of *Clostridium perfringens* present in District A-G and District B-H was 11.72% and 9.64%, respectively. The percentage of *Streptococcus* spp. was 11.27% in District A-G, but this organism was not found in District B-H (below the NONEUB control signal level).

### 3.5. Analysis of Endotoxin Levels in the TWM Hotspots

The levels of endotoxins in the hotspots of Market A after operations had been completed were as follows: District A-A, vegetables and fruits (409.64 ± 3.16 EU m^−3^) > District A-K, the entrance control (353.41 ± 4.62 EU m^−3^) > District A-I, live poultry and fresh raw meats (321.29 ± 2.57 EU m^−3^) > District A-G, livestock fresh raw meats (273.09 ± 1.52 EU m^−3^). The endotoxin levels in the hotspots of Market B after operations had been completed were as follows: District B-F, cooked food (979.92 ± 4.32 EU m^−3^) > District B-A, grains (682.73 ± 5.61 EU m^−3^) > District B-H, groceries (361.45 ± 2.62 EU m^−3^) > District B-K, the entrance control (224.90 ± 1.27 EU m^−3^).

## 4. Discussion

### 4.1. Influence of Impact Factors on the Bioaerosol Distribution in the Two Indoor TWMs

Since the characteristics of the two TWN buildings and the environmental conditions within the two indoor TWMs were similar (see Table 1 and Table S1), the differences in bioaerosol distributions can be attributed to the following factors. First, the presence of biological contamination in the two markets affected the distribution of indoor bioaerosols. The source of airborne bioaerosols in Market A before operations began was the biological residues from the slaughtering of poultry, such as chickens, by TWM staff and from the preparation of fresh raw meats, such as ground raw pork and beef, various raw chicken meat products, and raw fish fillets. Microorganisms were generated and increased in number as animal blood, excrement, and contaminated storage ice were generated by the market vendors. Since May 2013, Taiwan’s government has banned live livestock slaughter at TWMs. Another possible source of bioaerosols might have been the keeping of live poultry (chickens) in cages in Market A after the end of operations. Their feathers, urine, and excrement are continuous sources of biological contamination on the market floor over a long period of time. Since the hotspots of district A-H and district A-I were adjacent to the cooked food district (A-J) (see Figure 1), it seems likely that the cooked food might have been contaminated by the transfer of food poisoning bacteria via the air in this indoor facility. Moreover, the continuous activity of customers presents an important factor that affects the generation of biological contamination. This could also explain why the market entrances of District A-K and District B-K with the most staff and customers presented relatively high concentrations of BBs and FBs. Customers in Market B were still able to purchase some goods at particular stalls after operations had supposedly been finished, and this would have generated more BBs. This behavior meant that the chemical cleaning procedure undertaken in Market B led to a decrease in BBs of 30.8% only, while the agitated water spout system used in Market A led to a higher decrease in BBs of 64%. 

Secondly, the difference in occupancy number between the two markets is likely to have influenced the bioaerosol concentrations at these two sites. Market B has a higher occupancy area, 16.25 m^2^ on average for each vendor, and this could have increased the humidity and temperature, thus affecting the bioaerosol concentration present in this market compared with market A, which has an average area of 13.88 m^2^ for each vendor (see Table S1). However, the ventilation system at each market seems to play a very important role because it provides fresh air continuously, which help to control the concentration of bioaerosols present in the two TMWs. Traditional ventilation fans in Market A are operated continuously until they are destroyed and do not undergo a cleaning and maintenance process. Thus, high levels of BBs and FBs accumulate in the fans of Market A due to poor air exchange. On the other hand, all of the air-conditioner filters of Market B are washed or replaced once per month. Motors in the ventilation system also undergo a detailed check and maintenance process. Market B has relatively low levels of BBs, but these units might not be able to effectively remove FBs and PM_10_ under conditions of high relative humidity [38]. 

Third, the size distribution of bioaerosols is known to be affected by various biotic and abiotic factors including the microorganisms present, the environmental conditions, and human activity. The number of people visiting markets and the types of human activity carried out at markets usually show positive correlations with the level of BBs found there, but do not usually seem to affect the level of FBs found [28]. Previous studies have suggested that the most abundant fungal species found are the ones that produce small, light spores, and those that are less abundant seem to be the ones that produce fewer, bigger, and heavier spores, with the latter spores not becoming airborne as easily [21,22]. Since the products sold at the stalls in both indoor TWMs are, it seems likely that the different cleaning processes used by the two TWMs play important roles in altering the bacterial levels found during this study. The cleaning process used in Market A, which involves washing with an agitated waterspout, seems to result in a high proportion of SBP for both BBs (75%–78%) and FBs (63%–86%) (see Table 4). In Market A, vendors who washed their own stalls with an agitated waterspout were able to decrease the BB concentration by 37.7%, but in the process, the FB concentration increased by 2.43-fold (mostly SPB), which resulted in fungal SPBs having a wide distribution because they were raised into the air by air turbulence from the market’s ventilation fans and then they begin to settle on the stalls. The presence of stagnant water on the ground after washing might also help the release fungal SPB (e.g., spores) into the air due to evaporation. On the other hand, chemical sanitization on stall surfaces was used to remove airborne bacterial LBPs in Market B, and this might be the reason behind the 3-fold increase in the SBP/LBP ratio after operations had been completed. However, this professional sanitization process did not seem to have an obvious influence on the proportion of SBP FBs present. The ratio of fungal SPB decreased only slightly—from 82% before market operations began to 78% after market operations had been completed. These SPBs can be inhaled directly into the human respiratory system and are known to cause acute inflammation of the lungs [13,17]. A high SPB concentration will create a high health risk among market staff and lead to disease at indoor TWMs. For a summary of airborne bioaerosols in the two indoor TWMs compared in this study, see Table S3. 

### 4.2. Bacterial Species and Their Possible Pathogenicity in Indoor TWMs

The bacterial community present in a given market depends on the goods being sold at a traditional market and/or the particular environment present within that traditional market. *Alcaligenes* spp., which are members of β-Proteobacteria, and *Enterobacter* spp., which are members of *γ*-Proteobacteria, as well as *Corynebacteria* spp., *Micrococcus* spp., and *Acinetobacter* spp., which are members of Actinobacteria, together with *Bacillus* spp., which are members of Firmicutes, have been frequently identified as present in border markets in Thailand [12]. The number of Gram-negative bacteria found in the fruit market area has been reported to be a good indicator of inadequate ventilation and overcrowding in traditional markets [39]. Gram-positive spore-forming anaerobic *Clostridium* spp. are bacteria that can normally be found in the intestines of both humans and livestock. In addition, *Pseudomonas* spp. are often found as opportunistic pathogens in humans and animals and can also be isolated from spoiled food [40,41]. 

*Pseudomonas* spp. are very versatile γ-Proteobacteria and include species that are important in aerobiology. *Pseudomonas* spp. can be pathogenic to plants, animals, and humans, and they are also able to release endotoxins. “*Pseudomonas* infections” are defined as illnesses that are caused by *Pseudomonas* spp. These species include *Pseudomonas dermatitis* and otitis externa, and during the courses of these diseases, the organisms are able to invade the human lungs as well as infecting the skin. *Pseudomonas* spp. are also associated with the spoilage of meat during which they cause off-odors, off-flavors (aldehydes, ketones and esters), discoloration, and gas production [40]. The high level of *Pseudomonas* spp. present in TWMs is probably related to the presence of raw meat. Previous studies have indicated that *P. fluorescens, P. putida, P. lundensis, P. migulae*, and *P. fragi* strains can be isolated from various different types of meat, namely beef, poultry, pork, rabbit, and lamb, as well as from seafood (fish); such sources include both fresh and “spoiled” samples that have been stored using ice for several days [30,41]. A high prevalence of *P. aeruginosa* suggests post-processing contamination and the presence of meat that is prone to spoilage. *P. aeruginosa* strains have been isolated from raw meats, including chicken, pork, buffalo, and goat, being sold in the open air without adequate temperature control [42,43]. 

The presence of *C. perfringens* poses a high risk to the health of customers who shop at indoor markets. Cooked fresh food sold in such markets can become contaminated with this pathogen; such foods include meat dishes, poultry dishes, soups, gravy, and sauces. All of these allow the rapid growth of *C. perfringens*. Illness results from the entry of spores and/or enterotoxins into the intestines, and this can cause watery diarrhea, nausea, vomiting, abdominal pain, and fever. *C. perfringens* can also cause serious infection of the human throat as well as infections at wound sites [44]. The source of *C. perfringens* is likely to be the keeping of live poultry at TWMs as well as the presence of chopped fresh raw meat at market stalls during market operation. The concentration of *C. perfringens* is likely to increase rapidly and be released into the indoor air of TWMs at room temperature, because suitable conditions for this bacteria’s incubation are present in many markets.

*Streptococcus* spp. are facultatively anaerobic Gram-positive sphere-shaped bacteria that are commonly found on the epidermis and in the respiratory tracts of warm-blooded animals; they may be pathogenic under certain circumstances. For example, *S. suis*, which is a significant pathogen that affects pigs, was isolated from 6.1% of raw pork meat samples obtained from tongues, tonsils, bone, and tails in three out of six wet markets in Hong Kong [31]. Serotype 2 strains have been reported to be highly virulent and are often prevalent in intensive swine rearing areas worldwide; they are known to cause serious disease outbreaks in both pigs and humans. *S. pyogenes* causes Streptococcal pharyngitis (strep throat) in the upper respiratory tract, impetigo of the skin, sore throat, erysipelas, necrotizing fasciitis, and acute bacterial endocarditis. *S. pneumoniae* can also cause pneumonia and otitis media. In this study, the possible source of *Streptococcus* spp. was the fresh livestock meat district of Market A. It is here that raw pork products are retailed, and it is highly likely that there is frequent cross-contamination during post-slaughter processing, distribution, storage, and display.

### 4.3. A Comparison of the Endotoxins of Gram-Negative Bacteria Found in Two Indoor TWMs

The endotoxin levels were proportional to the presence of BBs, because these are the major source of endotoxins; endotoxins are released from the cell walls of Gram-negative bacteria, such as α-Proteobacteria, β-Proteobacteria, and γ-Proteobacteria. These findings suggest that the cleaning process seems to be able to reduce the level of endotoxins released by Gram-negative BBs. The highest level of endotoxins was found in District A-A, and this can be ascribed to the increased level of BBs present, from 3.18 × 10^2^ CFU m^−3^ before market operations commenced to 9.54 × 10^2^ CFU m^−3^ after market operations finished (see Figure 3 and Table S2). The feeding of live poultry in cages is highly likely to increase indoor bioaerosol and endotoxin levels [45]. The concentration of endotoxins found in the entrance of Market A (District A-K, control) was 353.41 EU m^−3^ and there was a relatively high concentration of BBs, which can be ascribed to the presence of iron cages that were stacked on the ground in this area. These cages had been used to raise poultry and did not seem to have been cleaned; this meant that significant amounts of biological residue, such as chicken feathers and chicken excrement, remained attached to these cages. It is important to note that high endotoxin concentrations and high concentration of *P. aeruginosa* have been found previously in the bacterial bioaerosols present in live poultry markets [46]. Moreover, there are two possible reasons why the endotoxin level in Market B hotspots was higher than in Market A. Firstly, chemical sanitization was used in Market B to provide an effective cleaning process after market operations had been completed; this would have reduced the concentration of BBs present. During this process, the cell walls of the Gram-negative bacteria would have been destroyed and endotoxins generated. These would have then been released into the indoor air. In addition to the above risks to market staff, some customers remained in the cooked foods district (District B-F) after market operations had finished, and there might have also been a significant health risk for these individuals. 

Endotoxin exposure for different time courses can result in the body’s inflammatory response being increased significantly [47]. Major bioaerosol-related diseases associated with airborne endotoxins include asthma, asthma-like syndrome, and a number of other respiratory diseases such as chronic bronchitis, organic dust toxic syndrome, allergic rhinitis, leukopenia, the Schwartzman reaction, endotoxemia, and anaphylactic shock 48]. Indoor workers who inhale these endotoxins can suffer from acute fever or flu/cold-like symptoms, and eventually, the endotoxins might affect the normal functioning of the lungs [19,48]. As a result, the threshold limit values for endotoxins have been widely discussed, particularly in indoor workplaces [19]. “No observed effect levels (NOEL)” for endotoxins using various health endpoints have been proposed in The Netherlands by the Dutch Health Council, namely 50 EU m^−3^ (8-TWA) [49].

## 5. Conclusions

The concentrations of BBs and FBs present in Taiwan’s indoor TWMs vary and differ prior to the start of operations and after operations have finished. This study demonstrated current environmental conditions found in typical indoor TWMs in Taiwan even though the sampling was conducted in 2008. SPB made up 73% to 78% of the BBs and 63% to 86% of the FBs at the two indoor TWMs. The major source of bioaerosols in the market hotspots appeared to be the fresh raw meat at stalls selling livestock, poultry, and seafood. Live poultry raised in cages also appeared to be a significant source of high-risk biological contaminants. *Pseudomonas* spp. are quite dangerous bacteria; these were found to be widely distributed in the markets, and the concentration was related to the endotoxin levels present in the markets. Furthermore, *Streptococcus* spp. appeared to be present due to the pretreatment of fresh raw meat at stalls in Market A. The level of *Streptococcus* spp. may be a good biological indicator for detecting whether the raw meats sold in the market are fresh or not. Developing guidance on the safe operations of wet markets, which are an important source of affordable products and livelihood to many people in Taiwan and other regions across the world, is crucial. The different cleaning processes used after the operation of these indoor TWMs seems to affect the levels of bioaerosols present in these markets. Chemical sanitization was shown to remove 30.8% of airborne BBs and 19.2% of airborne FBs. However, this process resulted in more endotoxins, created by the lysis of Gram-negative bacteria, being released into the indoor air. An aggressive strategy is needed to reduce the concentration of bioaerosols present in indoor TWMs in order to protect the health of market staff. Based on our results, the city government’s office in charge of indoor TWMs should now be able to carry out effective prevention planning to reduce the biological risk to indoor TWM staff. The health risk to indoor TWM staff who are exposed to bioaerosols needs to be evaluated, since they are present for long periods of time in the market carrying out stall preparation, selling, and stall cleaning.

## Figures and Tables

**Table 1:** Districts of sampling locations within the indoor traditional wet markets (TWMs).

Market	Number of Vendors	Cleaning Process after Operations at the Market have Ended ^2^	Ventilation System during Operation	Districts No.	The Products Sold in Their Stalls	Sampling Locations Using Cartesian Coordinates (x; y) ^1^
A	151	Washing by agitated waterspouts	Ventilation fans	A-A	Vegetables and fruit	(19,625; 24,105)
				A-B	Other (jewelry)	(34,940; 29,115)
				A-C	Groceries	(4115; 24,105)
				A-D	Grains (include chicken eggs)	(4115; 39,140)
				A-E	General merchandise	(19,625; 39,140)
				A-F	Flowers	(25,785; 19,020)
				A-G	Fresh livestock raw meat	(31,470; 11,200)
				A-H	Fresh raw seafood including fish	(31,470; 5910)
				A-I	Live poultry and fresh raw meat	(31,470; 1,750)
				A-J	Cooked food	(8,000; 7040)
				A-K	Outdoor control (Entrance)	(21,000; 44,150)
				A-S	Origin (datum point) for the Cartesian coordinate plane used for the distance survey	(0; 0)
B	58	Chemical sanitization on stall surfaces by professional cleaning service ^3^	Air conditions	B-A	Grains (include chicken eggs)	(9750; 2200)
				B-B	Fresh poultry raw meat	(4000; 8800)
				B-C	Fresh raw seafood including fish	(15,500; 8800)
				B-D	Livestock and fresh raw meat	(24,465; 12,100)
				B-E	Vegetables and fruit	(13,500; 15,400)
				B-F	Cooked food	(35,250; 13,200)
				B-G	Flowers	(24,465; 9900)
				B-H	Groceries	(35,250; 6600)
				B-I	General merchandise	(29,250; 2200)
				B-J	Fresh livestock raw meat	(24,465; 6600)
				B-K	Outdoor control (Entrance)	(9750; 0.0)
				B-S	Origin (datum point) for the Cartesian coordinate plane used for the distance survey	(0.0; 0.0)

^1^: Unit: mm. ^2^: Cleaning activities in both markets are processing at the end of the operation. ^3^: Commercial bleach product used.

**Table 2:** Bioaerosols sampling times and environmental conditions at the two indoor TWMs.

Market	Experiments	Sampling Time	Temperature (Outdoors/Indoors) ^1^ (°C)	Relative Humidity (Outdoors/Indoors) ^1^ (%)	Wind speed (Outdoors/Indoors) ^1^ (m s^−1^)
A	Bioaerosol analysis before operations began	28 March 2008, 3:00–4:00 a.m.	16.9/24.5	53.0/55.8	1.9/0.0
	Bioaerosol analysis after operations had finished	28 March 2008, 3:00–4:00 p.m.	27.2/24.2	45.0/56.7	3.8/0.2
	Bacterial community and endotoxin analysis after operations had finished	24 June 2008, 3:00–5:00 p.m.	32.7/30.1	68.0/73.0	0.9/0.0
B	Bioaerosol analysis before operations began	30 March 2008, 3:00–4:00 a.m.	22.8/23.5	80.0/77.8	0.7/0.0
	Bioaerosol analysis after operations had finished	30 March 2008, 3:00–4:00 p.m.	18.1/21.1	84.0/73.0	2.6/0.0
	Bacterial community and endotoxin analysis after operations had finished	25 June 2008, 3:00–5:00 p.m.	32.3/31.4	67.0/68.5	3.8/0.0

^1^: Outdoor information on environmental conditions was acquired from the historical records on the weather website: https://e-service.cwb.gov.tw/HistoryDataQuery/; accessed on 1 January 2021.

**Table 3:** Fluorescence in situ hybridization (FISH) primers selected for this study.

Probe Name ^1^	Target Group	Target Site (rRNA Positions ^2^)	Probe Sequence from 5′ to 3′
EUB338 Mixed (I, II, III)	most Bacteria, *Planctomycetales Verrucomicrobiales*	16S (338–355)	I: GCT GCC TCC CGT AGG AGT II: GCA GCC ACC CGT AGG TGT III: GCT GCC ACC CGT AGG TGT
ALF1b	*Alphaproteobacteria,* some *Deltaproteobacteria*, *Spirochaetes*	16S (19–35)	CGT-TCG-(C/T)TC-TGA-GCC-AG
BET42a	*Betaproteobacteria*	23S (1027–1043)	GCC-TTC-CCA-CTT-CGT-TT
GAM42a	*Gammaproteobacteria*	23S (1027–1043)	GCC-TTC-CCA-CAT-CGT-TT
HGC69a	*Actinobacteria*	23S (1901–1918)	TAT-AGT-TAC-CAC-CGC-GT
LGC354A	*Firmicutes*	16S (354–371)	TGG-AGG-ATT-CCC-TAC-TGC
Ps56a	most true *Pseudomonas* spp.	23S (1432–1446)	GCT-GGC-CTA-GCC-TTC
STRC493	most *Streptococcus* spp. and some *Lactococcus* spp.	16S (493–511)	GTT AGC CGT CCC TTT CTG
Cp2	*Clostridium perfringens*	16S (199–218)	GCT-CCT-TTG-GTT-GAA-TGA-TG
NONEUB338	Control probe complementary to EUB338	-	ACT-CCT-ACG-GGA-GGC-AGC

^1^: from probeBases website [36]. ^2^: *Escherichia coli* numbering.

**Table 4:** Average concentrations and percentages of large particle bioaerosols (LPB) and small particle bioaerosols (SPB) in the two indoor TWMs.

Market	Bioaerosols	Experimental Conditions in the TWMs	Average Concentration ^1^ (CFUm^−3^)		Percentage of Bioaerosols (%)	
			LPB	SPB	LPB	SPB
**A**	Bacteria	Before operations	5.93 × 10^3^	2.12 × 10^4^	22	78
		After operations	2.39 × 10^3^	7.29 × 10^3^	25	75
	Fungi	Before operations	1.21 × 10^3^	7.42 × 10^3^	14	86
		After operations	7.71 × 10^3^	1.33 × 10^4^	37	63
B	Bacteria	Before operations	8.06 × 10^2^	2.54 × 10^2^	76	24
		After operations	2.01 × 10^2^	5.32 × 10^2^	27	73
	Fungi	Before operations	4.24 × 10^2^	1.91 × 10^3^	18	82
		After operations	4.13 × 10^2^	1.45 × 10^3^	22	78

^1^: based on the levels of all ten districts of selling goods.

**Table 5:** Percentage distribution of bacterial communities present in the two TWM hotspots after operations had finished.

Class	Bacterial Community	District A-G (%)	District B-H (%)
Domain	Most Bacteria, *Planctomycetales* *Verrucomicrobiales*	74.37 ± 4.90 ^2^ (12 ^1^)	70.87 ± 5.10 (14)
Phylum	*Alphaproteobacteria*, some *Deltaproteobacteria, Spirochaetes*	11.06 ± 2.87 * (15)	6.06 ± 1.48 * (9)
Phylum	*Betaproteobacteria*	34.75 ± 4.50 * (14)	17.11 ± 3.73 * (10)
Phylum	*Gammaproteobacteria*	15.62 ± 5.00 (14)	9.62 ± 1.55 (9)
Phylum	*Actinobacteria*	19.74 ± 3.07 (10)	18.30 ± 5.83 (14)
Phylum	*Firmicutes*	15.49 ± 3.66 (9)	18.52 ± 4.89 (9)
Genus	Most true *Pseudomonas* spp.	48.74 ± 1.67 * (9)	35.18 ± 4.88 * (13)
Genus	Most *Streptococcus* spp. and some *Lactococcus* spp.	11.27 ± 2.50 * (8)	3.30 ± 1.73 * (13)
Species	*Clostridium perfringens*	11.72 ± 2.06 (10)	9.64 ± 1.57 (10)
Control	-	5.39 ± 1.84 (8)	4.30 ± 1.42 (10)

*: A significant difference (*p* < 0.05) in the percentage distribution between the two districts; ^1^: Cell-count sample numbers; ^2^: the standard deviation for the percentage of bacterial communities based on cell-count samples.

## Supplementary Materials

The following are available online at https://www.mdpi.com/1660-4601/18/6/2945/s1, Table S1: Basic information about the two indoor TWMs investigated in this study. Table S2: Average total bioaerosol concentration in the two indoor TWMs. Table S3. A summary of airborne bioaerosol in two indoor TWMs compared in this study. Figure S1: The airborne-bioaerosol distribution in ten districts of indoor Market A: (**a**) BBs before operation; (**b**) BBs after operation; (**c**) FBs before operations; (**d**) FBs after operation. Figure. S2: The airborne-bioaerosol distribution in ten districts of indoor Market B: (**a**) BBs before operation; (**b**) BBs after operation; (**c**) FBs before operation; (**d**)FBs after operation.

## Abbreviations

ACI	Andersen Cascade Impactor
BBs	Bacterial Bioaerosols
FBs	Fungal Bioaerosols
FISH	Fluorescence in situ Hybridization
IAQS	Indoor Air Quality Standard
LAL	Limulus Amebocyte Lysate
LPBs	Large Particle Bioaerosols
SPBs	Small Particle Bioaerosols
TWMs	Traditional Wet Markets
