# Supplementary material for: An Investigation of Airborne Bioaerosols and Endotoxins Present in Indoor Traditional Wet Markets before and after Operation in Taiwan: A Case Study
Source: Int J Environ Res Public Health. 2021 Mar 13;18(6):2945. doi: 10.3390/ijerph18062945 (PMC7999578; doi:10.3390/ijerph18062945)
Supplement: Supplementary file 1 [file ijerph-18-02945-s001.pdf]

An investigation of airborne bioaerosols and endotoxins present in indoor traditional wet markets before and after operation in Taiwan: A case study

Supplementary Information

Table S1. Basic information about the two indoor TWMs investigated in this study.

Market	A	B
Age of the buildings investigated during this study (date)	26 (2008)	25 (2008)
Structural materials used for the Building's construction	Reinforced concrete with a ceramic-tile floor	Reinforced concrete with a ceramic-tile floor
Number of floors and operational area (m^2)	One, 2,097.08	One, 942.50
Number of vendors operating at the market	151	58
Occupancy area (m^2 per vender)	16.25	13.88

Table S2. Average total bioaerosol concentration in the two indoor TWMs.

Market	Bioaerosols	Experimental Conditions in the TWMs	Average Concentration [1] (CFU m^{-3})
A	Bacteria	Before operations	2.71×10^4
		After operations	9.68×10^3
	Fungi	Before operations	8.63×10^3
		After operations	2.09×10^4
B	Bacteria	Before operations	1.06×10^3
		After operations	7.34×10^2
	Fungi	Before operations	2.33×10^3
		After operations	1.86×10^3

[1]: based on the levels of all ten districts of selling goods in Table 1.

Table S3. A summary of airborne bioaerosol in two indoor TWMs compared in this study.

<table>
<tr><th colspan="2">Market</th><th>A</th><th>B</th></tr>
<tr><td rowspan="2">The size of major BBs with the percentage of total BBs</td><td>Before operations</td><td>SPBs (78.15%)</td><td>LPBs (76.00%)</td></tr>
<tr><td>After operations</td><td>SPBs (75.32%)</td><td>SPBs (72.55%)</td></tr>
<tr><td rowspan="2">Hotspots of BBs with the percentage of total BBs</td><td>Before operations</td><td>A-G (36.08%), A-H (23.34%), A-I (34.95%)</td><td>B-H (28.00%), B-A (25.00%), B-E (14.00%)</td></tr>
<tr><td>After operations</td><td>A-G (24.74%), A-H (21.46%), A-I (29.61%)</td><td>B-E (23.11%), B-A (14.45%), B-D (14.45%)</td></tr>
<tr><td colspan="2">Dominant bacterial community at the hotspot (Phylum/Genes)</td><td>β-Proteobacteria 34.75±4.50%/Most true Pseudomonas spp. 48.74±1.67%</td><td>Firmicutes 18.52±4.89%/ Most true Pseudomonas spp. 35.18±4.88 %</td></tr>
<tr><td rowspan="2">The size of major FBs with the percentage of total FBs</td><td>Before operations</td><td>SPBs (86.00%)</td><td>SPBs (81.82%)</td></tr>
<tr><td>After operations</td><td>SPBs (63.23%)</td><td>SPBs (77.78%)</td></tr>
<tr><td rowspan="2">Hotspots of FBs with the percentage of total FBs</td><td>Before operations</td><td>A-G (16.46%), A-H (5.28%), A-I (64.99%)</td><td>B-C (10.91%), B-D (10.91%), B-F (12.73%), B-H (11.82%)</td></tr>
<tr><td>After operations</td><td>A-G (18.36%), A-H (38.44%), A-I (32.52%)</td><td>B-E (13.10%), B-H (17.09%), B-I (10.82%), B-J (14.24%)</td></tr>
<tr><td colspan="2">Impact factors on the bioaerosol distribution</td><td>slaughtering poultry, live poultry in cages, agitated waterspout of clean process, occupancy number, ventilation fans of ventilation system</td><td>continuing activity of customers after operation, chemical sanitization of clean process, air conditions of ventilation system</td></tr>
</table>

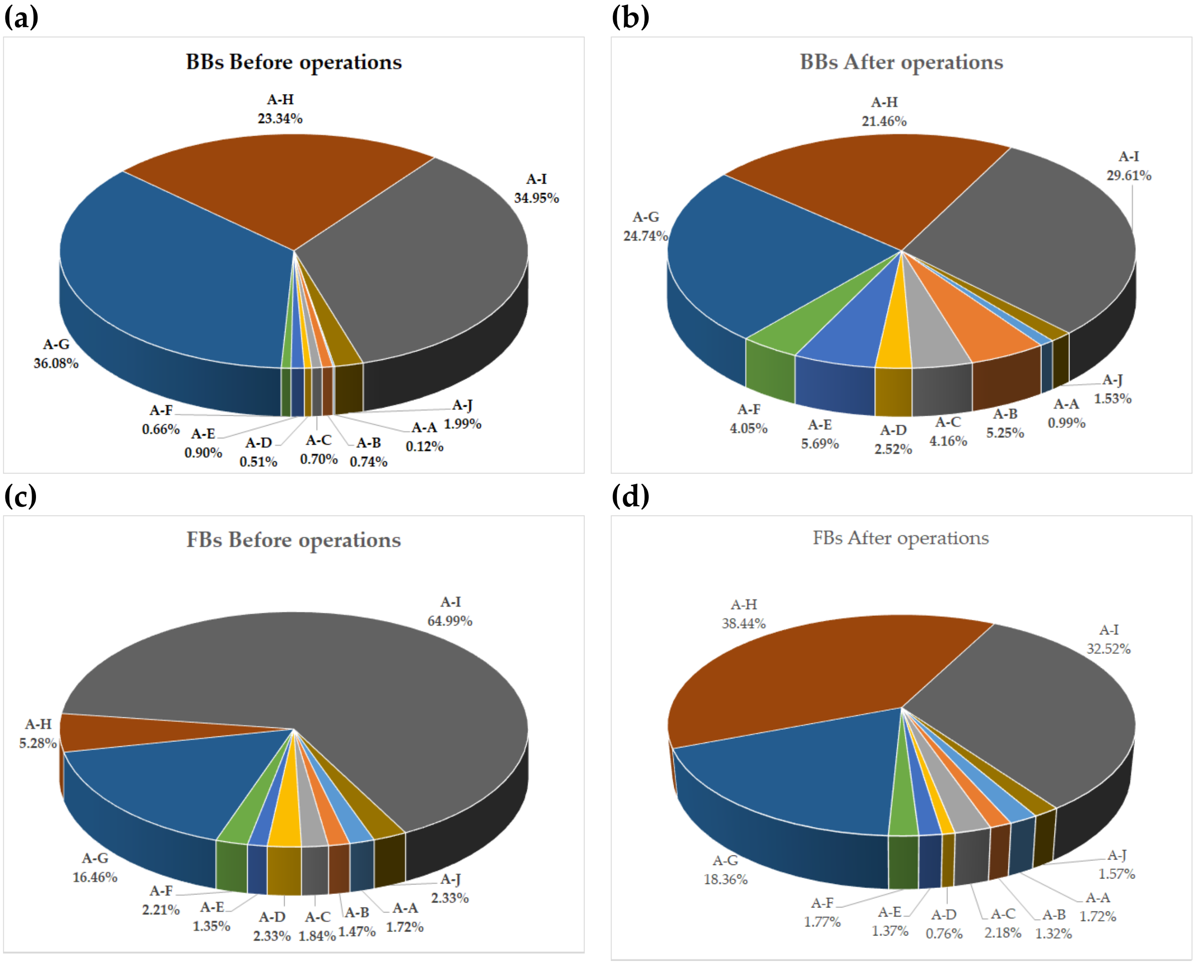

Figure. S1 The airborne-bioaerosol distribution in ten districts of indoor Market A: (**a**) BBs before operation; (**b**) BBs after operation; (**c**) FBs before operations; (**d**) FBs after operation.

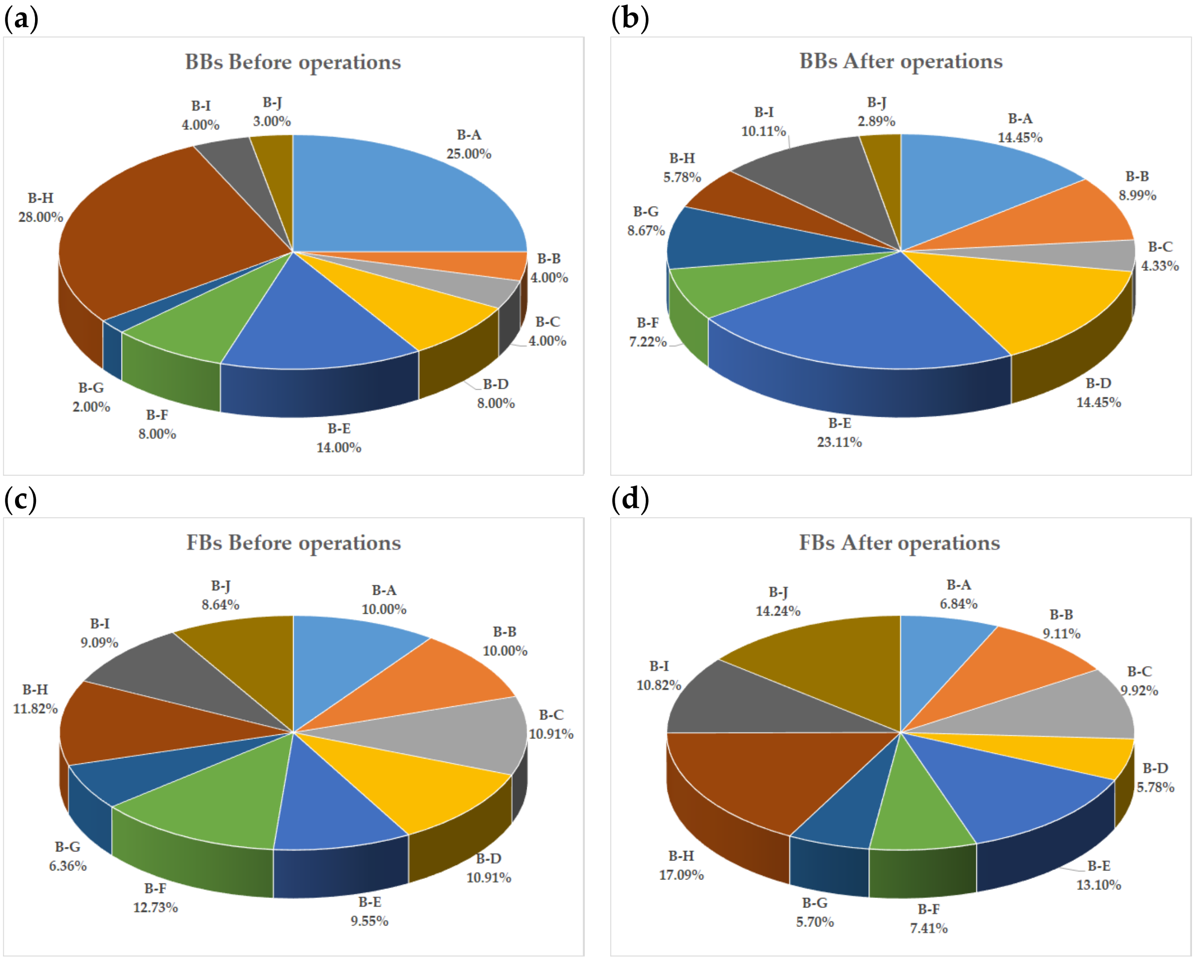

Figure. S2 The airborne-bioaerosol distribution in ten districts of indoor Market B: (a) BBs before operation; (b) BBs after operation; (c) FBs before operation; (d)FBs after operation.
